# Arrhythmogenic Inflammatory Cardiomyopathy in Autoimmune Rheumatic Diseases: A Challenge for Cardio-Rheumatology

**DOI:** 10.3390/diagnostics9040217

**Published:** 2019-12-10

**Authors:** Sophie I. Mavrogeni, George Markousis-Mavrogenis, Constantina Aggeli, Dimitris Tousoulis, George D. Kitas, Genovefa Kolovou, Efstathios K. Iliodromitis, Petros P. Sfikakis

**Affiliations:** 1 Onassis Cardiac surgery Center, 17674 Athens, Greece; georgemm32@gmail.com (G.M.-M.); genovefa@kolovou.com (G.K.); 2 First Cardiac Clinic, Hippokration University Hospital, 17674 Athens, Greece; dina.aggeli@gmail.com (C.A.); drtousoulis@hotmail.com (D.T.); 3 Arthritis Research UK Epidemiology Unit, Manchester University, Manchester M13 9PT, UK; gkitas@hygeia.gr; 4 Second Cardiac Clinic, Attikon University Hospital, 17674 Athens, Greece; iliodromitis@yahoo.gr; 5 First Department of Propeudeutic and Internal medicine, Laikon Hospital, Athens University Medical School, 17674 Athens, Greece; sfikakisp@gmail.com

**Keywords:** cardiovascular magnetic resonance, positron emission tomography, autoimmune myocarditis, autoimmune inflammatory cardiomyopathy, autoimmune rheumatic diseases

## Abstract

Ventricular arrhythmia (VA) in autoimmune rheumatic diseases (ARD) is an expression of autoimmune inflammatory cardiomyopathy (AIC), caused by structural, electrical, or inflammatory heart disease, and has a serious impact on a patient’s outcome. Myocardial scar of ischemic or nonischemic origin through a re-entry mechanism facilitates the development of VA. Additionally, autoimmune myocardial inflammation, either isolated or as a part of the generalized inflammatory process, also facilitates the development of VA through arrhythmogenic autoantibodies and inflammatory channelopathies. The clinical presentation of AIC varies from oligo-asymptomatic presentation to severe VA and sudden cardiac death (SCD). Both positron emission tomography (PET) and cardiovascular magnetic resonance (CMR) can diagnose AIC early and be useful tools for the assessment of therapies during follow-ups. The AIC treatment should be focused on the following: (1) early initiation of cardiac medication, including ACE-inhibitors, b-blockers, and aldosterone antagonists; (2) early initiation of antirheumatic medication, depending on the underlying disease; and (3) potentially implantable cardioverter–defibrillator (ICD) and/or ablation therapy in patients who are at high risk for SCD.

## 1. Introduction

Ventricular arrhythmia (VA) is associated with high morbidity and mortality [1]. Specifically, malignant arrhythmia is the leading cause of sudden cardiac death (SCD) in Western countries, with >1000 SCDs occurring every day in the United States [1]. Although structural heart diseases, particularly coronary artery disease (CAD) and heart failure (HF) [2], are the main underlying causes of SCD, structural changes were not identified at the postmortem examination in 5–15% of patients, a percentage increasing up to 40% in patients under 40 years old [1].

VA is also commonly associated with autoimmune rheumatic diseases (ARDs). Seferovic et al. [3] described rhythm/conduction disturbances and SCD in ARDs. Myocardial scar due to ischemic or nonischemic heart disease is the main cause of structural disease in ARDs [4]. Myocardial inflammation, either isolated or as a part of the general inflammation, is another important cause of VA in ARDs [4].

The term “arrhythmogenic inflammatory cardiomyopathy” (AIC) was recently proposed and includes a group of patients with nonischemic cardiomyopathy (NICM), who were referred for management of VA and were found to have evidence of active myocardial inflammation. Our aim in this review is to describe the profile of AIC in patients with ARD, suggest a diagnostic algorithm, and propose a “cardiorheumatic” therapeutic approach.

## 2. Pathophysiology of AIC in ARDs

### 2.1. Fibrotic Substrate

Structural heart disease includes all causes of underlying myocardial fibrotic substrate (scar). The most common heart disease in ARDs leading to fibrotic substrate is ischemic cardiomyopathy (ICM)/heart failure (HF), which is caused by atherosclerotic coronary artery disease [5]. However, NICM that may lead to AIC represents another large group of AICD patients with primary cardiac dysfunction and normal coronary vessels. Specifically, in ARDs, dilated cardiomyopathy with normal coronary arteries can be found in rheumatoid arthritis (RA); vasculitis and systemic lupus erythematosus (SLE); myocarditis in RA, SLE, systemic sclerosis (SSc), and vasculitis; diffuse subendocardial fibrosis in small vessel vasculitis and SSc; and, finally, infiltrative myocardial disease in sarcoidosis and amyloidosis [5].

Re-entry is the most common mechanism responsible for ventricular tachycardia (VT) in AIC and is due to the presence of anisotropic conduction occurring in a mixture of healthy myocardial tissue interspersed with scar tissue. These different types of tissue also have different conduction and refractory period properties. The post-myocardial infarction scar is a complex heterogenous mixture of viable myocardial cells interspersed with fibrotic tissue [6]. In NICM, scar is also a combination of interstitial and replacement fibrosis, myocyte atrophy/hypertrophy, and myofiber disarray interspersed with normal myocardial cells, leading to regions characterized by abnormal conduction that may lead to VT development [7].

### 2.2. Inflammatory Substrate

The role of cardiac inflammation as a causative factor of AIC in autopsy/biopsy-proven inflammatory cell infiltration in ARDs is well documented [8,9,10,11,12]. It is also clear that systemically released autoantibodies and cytokines can be per se arrhythmogenic, regardless of the presence of histologic alterations in the myocardium [13,14,15]. Several arrhythmogenic autoantibodies targeting calcium, potassium, or sodium channels in the heart have been identified, and therefore the term autoimmune cardiac channelopathies was proposed [16]. Furthermore, there is evidence that the inflammatory cytokines, mainly tumor necrosis factor (TNF)-a, interleukin-1, and interleukin-6, can modulate the expression and function of ion channels, both by directly acting on cardiomyocytes [17] and/or inducing systemic effect [17]. These largely overlooked factors are potentially involved in several unexplained arrhythmias/SCD in ARDs, without any known genetic factor.

Cardiac or systemic inflammation may promote QTc-interval prolongation via cytokine-mediated intracellular pathways, increasing the risk for SCD [18]. This is supported by several studies in patients with inflammatory heart diseases, ARDs, and infections, and even in apparently healthy subjects with low-grade chronic systemic inflammation, such as obesity [19,20,21,22,23]. It seems that inflammation, regardless of its origin, represents a risk factor for long QTS (LQTS) and life-threatening VA. Moreover, in patients with elevated CRP, due to various inflammatory conditions, QTc prolongation is common. Furthermore, CRP, TNF-a, and interleukin-6 decrease associates with a significant QTc shortening [24]. It has been documented that inflammatory cytokines and TNF-a prolong ventricular action potential duration (APD) by inducing dysfunction of cardiac ion channels, particularly K^+^ channels [18].

### 2.3. Cardiac Channelopathy due to Specific Autoantibodies

All these disorders are caused by the dysfunction of specific cardiomyocyte ion channels, leading to a disruption of the cardiac action potential (AP) [25]. The electric abnormalities lead to increased susceptibility for arrhythmias, syncope, seizures and/or SCD. Two LQTS-induced autoimmune channelopathies have been identified, and both are associated with autoantibodies cross-reaction with specific K^+^ channels. Anti-Ro/SSA antibodies can be the cause of a novel form of acquired LQTS via cross-reaction and blockade of the hERG-K^+^ channel [26] Anti-Ro/SSA antibodies are among the most frequently detected autoantibodies in several ARDs and also in healthy populations [27,28]. Patients and their offspring with anti-Ro/SSA-positive ARDs commonly present QTc prolongation, correlating with autoantibody levels (particularly anti-Ro/SSA 52-kD) and complex VA [27]. Although pathophysiologic studies are still missing, LQTS seems to be the result of an autoantibody-dependent inhibition via direct channel binding [29,30]. Nevertheless, because signs of myocarditis are present in some patients suffering from myasthenia gravis with positive anti-Kv1.4 [29,30], we may assume that both inflammatory mechanisms and structural heart changes may contribute to the pathogenesis of electric alterations.

## 3. AIC in ARD Patients

Although multicenter studies about VA in ARDs are still missing, there are some publications presenting evidence of AIC in various ARDs. In rheumatoid arthritis (RA), the most common cause of SCD is atherosclerotic coronary artery disease (CAD) that may lead to acute coronary syndrome and VT [3]. Additionally, VT was detected as a consequence of therapeutic interventions such as low dose methotrexate administration [31] or infliximab infusion [32]. Finally, giant-cell myocarditis (GCM), a rare but frequently fatal cardiac inflammation of unknown origin, characterized by degeneration and necrosis of myocardial fibers, can also be presented as VT during the course of RA and independently of some response to immunosuppressive treatment or of the development of heart failure (HF), which contributes to poor prognosis [33].

In systemic lupus erythematosus (SLE), although supraventricular tachycardia is the most common finding, VT is not uncommon and is mainly due to CAD [3]. Chloroquine plays a protective role in the unexpected high rate of cardiac arrhythmias and conduction disturbances observed in SLE [34]. Acute myocarditis and VA can also be documented at the initial presentation of SLE in children [35]. Early diagnosis of the disease with a combination treatment for HF, arrhythmias, and immunosuppression may lead to a better prognosis [35]. Acute myocarditis in SLE may present with VT as a first manifestation [35]. LQTS with atrioventricular block and VT can be also developed in neonates of mothers with SLE [3]. Finally, the chronic use of antimalarial drugs may also lead to VT [36]. In the era of implanted devices, an implantable defibrillator device (ICD) is a necessary adjunct to medical treatment in ARDs with lethal arrhythmias [37].

In systemic sclerosis (SSc), non-sustained VT was described in 7–13%, while SCD was reported in 5–21% of unselected SSc patients [3]. Recently, SAnCtUS, the only multicenter study evaluating VA in SSc, found that a CMR score combining inflammatory and fibrotic indices can provide information regarding VA prediction in SSc patients [38]

VT and SCD can be also assessed during polymyositis (PM) and dermatomyositis (DM), although their incidence has been poorly defined [39,40,41,42].

## 4. Clinical Manifestations of AIC in ARDs

There is a great spectrum of clinical manifestations of AIC in ARDs, starting from clinically silent presentation and extending to overt acute or chronic HF [43,44,45,46,47]. However, the most common manifestation of AIC in ARDs remains HF that may either have an oligo-asymptomatic onset or a rapidly progressive emerging course, leading to cardiogenic shock. In these cases, only the implantation of a mechanical circulatory support device or urgent heart transplantation can be life-saving. If the patient survives the acute phase, a significant improvement or even a complete recovery of LV systolic function with excellent long-term prognosis may occur [48].

## 5. Diagnostic Algorithm of AIC in ARDs

The diagnostic algorithm of AIC still remains a real challenge for the clinician. Electrocardiogram (ECG), echocardiogram (Echo), positron emission tomography imaging (PET), and cardiovascular magnetic resonance (CMR) are important for the diagnosis of AIC. ECG can show specific and nonspecific findings, including any type of arrhythmias, changes of PQ and ST interval, prolongation of QRS complex, and the presence of Q waves. Some findings, especially the presence of rhythm disorders, i.e., VT or atrioventricular block, may be suggestive of giant-cell myocarditis and/or cardiac sarcoidosis.

The baseline diagnostic modality is conventional echocardiography (ECHO). There are no typical ECHO findings that can support the diagnosis of AIC. Both global and regional kinetic disorders of the left or right ventricle, diastolic dysfunction, left ventricle hypertrophy, and pericardial effusion should be taken under serious consideration. Newer echocardiographic modalities, like 2D–3D speckle tracking imaging derived by transthoracic echocardiography, should be offered more accurate data for the early diagnosis of the myocardial involvement. Furthermore, several studies [49,50] supported that the early progression of left ventricular systolic dysfunction was demonstrated by global longitudinal strain (GLS) and not by left ventricular ejection fraction. However, even a normal echo cannot rule out the diagnosis. Its main value lies rather in excluding other causes of the symptoms (valvular or pericardial disease, aortic dissection) and also in risk stratification based on evaluation of left ventricle systolic dysfunction [51,52].

Although clinical, ECG, and conventional ECHO evaluation is the very first approach in AIC, new advances of echocardiographic imaging techniques such as 2D or 3D global longitudinal strain (GLS) can detect early myocardial involvement and select patients for further evaluation. However, only positron emission tomography imaging (PET) and cardiovascular magnetic resonance (CMR) can provide reliable diagnostic information about inflammation/scar by performing tissue characterization.

The proposed diagnostic algorithm for AIC using PET includes the following [43]: (1) nonischemic cardiomyopathy with left ventricular ejection fraction (LVEF) of <50%; (2) documented VA including monomorphic/polymorphic VT, ventricular fibrillation, or frequent premature ventricular contractions; (3) patchy focal or focal on diffuse fluorodeoxyglucose (FDG) uptake on PET imaging.

Diffuse uptake was excluded from the diagnostic algorithm, because the inadequate fasting/physiologic uptake may reduce its specificity [43]. AIC can be further categorized according to perfusion/metabolism mismatch by inflammation in the presence of scar (late AIC) and in the absence of scar (early AIC), with or without extra cardiac involvement.

**Figure 1:**
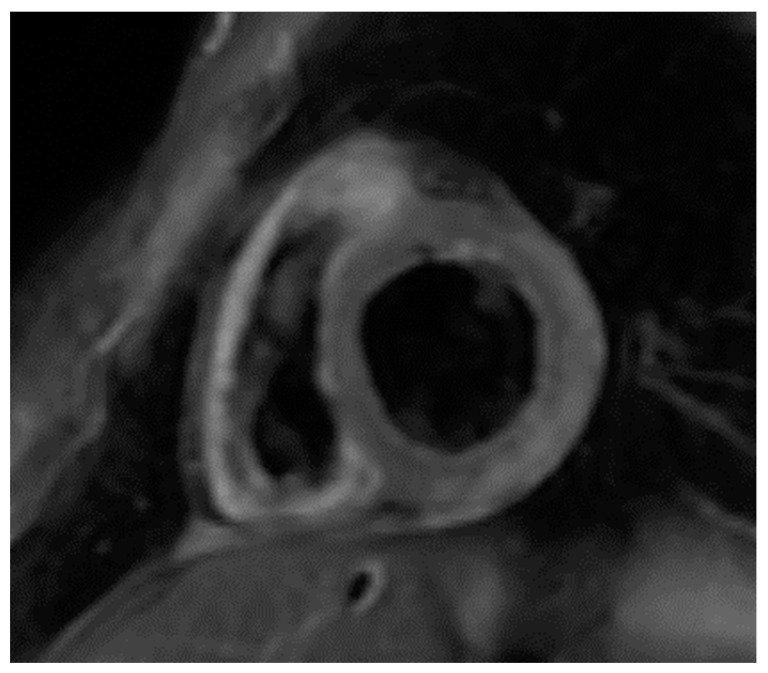
Short axis T2 image showing edema (bright area) in the lateral wall of LV of a patient with SLE presented with VT.

**Figure 2:**
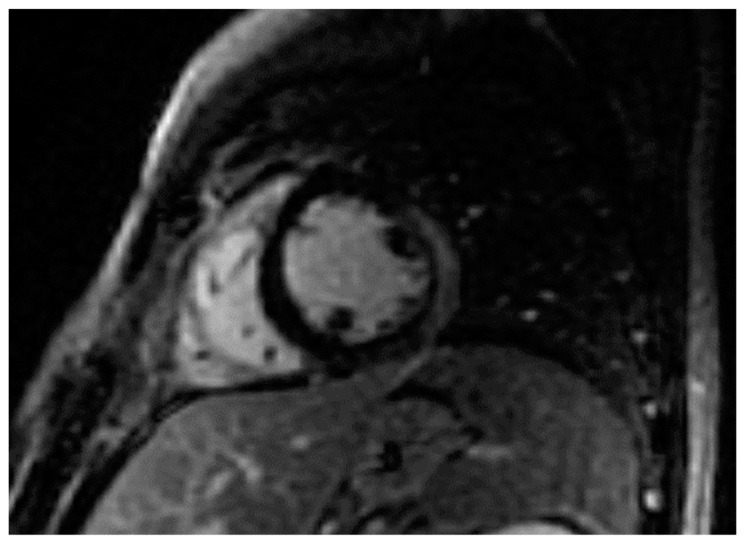
Short axis LGE image showing subepicardial fibrosis (bright area) in the lateral wall of LV of the same patient.

Currently, there is no specific algorithm for AIC using CMR. However, there is a diagnostic algorithm, by an expert consensus, using CMR for assessment of acute and chronic inflammatory cardiomyopathy [45]. According to this, in patients with suspected acute or active myocardial inflammation, apart from Lake Louise Criteria [44], the use of parametric mapping techniques (T2, native T1, post contrast T1, and ECV mapping) is recommended. While each parameter may indicate myocardial inflammation, the authors proposed that CMR provides strong evidence for myocardial inflammation, with increasing specificity, if the CMR scan demonstrates the combination of myocardial edema with other CMR markers of inflammatory myocardial injury (Figure 1 and Figure 2). This is based on at least one T2-based criterion (global or regional increase of myocardial T2 relaxation time or an increased signal intensity in T2-weighted CMR images), with at least one T1-based criterion (increased myocardial T1, extracellular volume, or late gadolinium enhancement).

While having both a positive T2-based marker and a T1-based marker will increase specificity for diagnosing acute myocardial inflammation, having only one (i.e., T2-based or T1-based) marker may still support a diagnosis of acute myocardial inflammation in an appropriate clinical scenario, but with less specificity [45]. Specifically, for ARDs, a combination of edema–fibrosis imaging was also proposed to describe the pathophysiologic background of AIC [46,47]. A schematic presentation of advantages and disadvantages of Echo, PET, and CMR is presented in Table 1.

Nearly 50% of patients referred with unexplained cardiomyopathy and VA demonstrate ongoing focal myocardial inflammation on FDG PET. These data suggest that a significant proportion of patients labeled “idiopathic” may have occult AIC, which may benefit from early detection and immunosuppressive medical therapy [53].

Coronary angiography (CA) has a role only for the exclusion of coronary artery disease.

Endomyocardial biopsy (EMB) is an invasive approach that can provide tissue for histopathologic evaluation and is still considered to be the gold standard to diagnose myocarditis and/or inflammatory cardiomyopathy. Unfortunately, the patchy distribution of the disease does not allow an accurate diagnosis [44]. Therefore, while its specificity is excellent, sensitivity is poor due to many factors, including sampling errors and lack of agreement between the specialists regarding the specimen interpretation.

## 6. Role of Troponin

Cardiac troponins are specific for myocardial cell damage, but not myocardial infarction, and can be elevated in numerous other disease states, such as renal failure, sepsis, pulmonary embolism, and cardiac injury after chemotherapy, such as with trastuzumab and doxorubicin. In these cases, myocardial injury can be diagnosed independently of myocardial ischemia. Therefore, in these cases, its clinical significance in both diagnosis and prognosis remain questionable. In a study of 215 patients with increased troponin and normal coronary arteries, the spectrum of disease identified by CMR was myocarditis (32%), small area infarction (22%), nonischemic cardiomyopathy (20%), and stress cardiomyopathy (9.3%). This study documents the great clinical value of CMR as a tool to identify various pathophysiologies in patients with an increase of troponin [54]

A current meta-analysis showed the moderate sensitivity and specificity of F-18 FDG PET or PET/CT for diagnosis of cardiac sarcoidosis (CS). The presence of combined myocardial perfusion imaging could improve diagnostic accuracy of F-18 FDG PET or PET/CT for diagnosis of CS. At present, the literature regarding the use of F-18 FDG PET for detection of CS remains limited; thus, further large multicenter studies would be necessary to substantiate the diagnostic accuracy of F-18 FDG PET for the diagnosis of CS [55].

While CMR and FDG-PET provide complementary information in CS evaluation, current guidelines do not recommend which advanced imaging modalities are essential in suspected CS and, if so, which modality should be performed first. The utility of hybrid imaging combining both advanced imaging modalities in a single scan is currently being explored, although not yet widely available [56].

## 7. Treatment of AIC in ARDs

The treatment of AIC in ARDs should include both a cardiologic and a rheumatologic approach. The cardiologic approach includes a pharmacologic and an interventional aspect.

### 7.1. Cardiologic Pharmaceutical Approach

#### 7.1.1. Beta Blockers Administration

Beta blockers are of great value in the management of HF with reduced ejection fraction (HFrEF). Furthermore, carvedilol was found to be cardioprotective, due to suppression of inflammatory cytokines, while both metoprolol and propranolol were not [57]. Beta blockers also have an independent advantage for the survival of patients with VA who don’t already have an ICD as it is the majority of AIC patients [58].

#### 7.1.2. ACE Inhibitors and/or ARBs

Angiotensin-converting enzyme (ACE) inhibitor and angiotensin-receptor blocker (ARB) treatment is well established in HFrEF. Their early initiation is of great help to minimize the ventricular remodeling and improve survival.

### 7.2. Aldosterone Antagonists

Aldosterone antagonists play an important role in the management of HFrEF patients, irrespective of the etiology of primary myocardial disease. Although there are small mouse model studies supporting the anti-inflammatory benefits in viral myocarditis, there have not been studies in AIC patients.

### 7.3. Cardiologic Interventional Approach

#### 7.3.1. Implantable Cardioverter–Defibrillator Therapy

Implantable cardioverter–defibrillator (ICD) treatment is currently the cornerstone of HFrEF management since the multicenter automatic defibrillator

Implantation Trials (MADIT and MADIT-II) and Sudden Cardiac Death in Heart Failure Trial (SCD-HeFT) had demonstrated reduced mortality over 1-to-5-year follow-ups [59,60,61]. However, in these studies, a combination of both ICM and NICM was included, and therefore it was difficult to assess the potential benefit for AIC. Recently, the Danish Study to Assess the Efficacy of ICDs in Patients with Nonischemic Systolic Heart Failure on Mortality (DANISH) study of 556 patients with NICM and symptomatic HF found that there was an insignificant reduction in the primary outcome of all-cause mortality, but with a statistically significant reduction in SCD with ICD over controls [62]. However, in this study the pathophysiologic background of various NICM were not taken into consideration. For example, giant-cell myocarditis, a very rare form of AIC, is usually fatal within six months without heart transplantation. In this case, although ICD therapy may prevent SCD, it cannot change the long-term survival of the underlying disease. However, other diseases such as cardiac sarcoidosis can be improved by a combination of autoimmune treatment and ICD implantation [63]. The special characteristics of various ARDs should motivate multicenter studies to create specific disease-based algorithms about the indications of ICD implantation in this target group. Finally, the recent experience from new imaging modalities, such as CMR and PET, in combination with electrophysiologic studies, should be taken under serious consideration and used as a guide for decision making [64,65,66,67].

#### 7.3.2. Ablation Therapy

The role of radiofrequency ablation in patients with AIC is not well evaluated. It is known that there is a clear discordance regarding the long-term effect of ablation in ICM and NICM. This difference was attributed to a lack of modifiable substrate (scar) in NICM patients that may have a combination of scar-based re-entrant VT and functional VT not directly related to myocardial scar or fibrosis. Kumar et al. investigated the characterization of substrate and outcomes after ablation in 435 patients with cardiac sarcoidosis. They identified that the mechanism of cardiac sarcoid-related VT is likely to be the result of re-entry involving confluent regions of scarring in the RV endocardium and epicardium along with patchy LV endocardial scarring affecting the basal septum, anterior wall, and perivalvular regions. Catheter ablation was able to terminate VT storm and >1 inducible VT in the majority of patients, resulting in reduction in ICD shocks. However, recurrences were common, and the failure to abolish all VT episodes was attributed to intramural circuits [68]. It seems that VA in these patients is the common point of myocardial scar and concurrent inflammation. Unfortunately, the only experience we have comes from cardiac sarcoidosis. However, other ARDs, such as inflammatory myopathies and systemic sclerosis, are also prone to AIC and the risk of VA/SCD. Therefore, since these patients are at lifelong risk for SCD, a new pharmacologic and interventional algorithm should be proposed.

#### 7.3.3. Pharmacologic Treatment

Amiodarone remains the most efficacious therapy for the reduction of appropriate and inappropriate shocks in patients with ICD. No therapy resulted in mortality reduction, but amiodarone showed a trend toward increased mortality [69].

### 7.4. Rheumatologic Pharmaceutical Approach

Rheumatologic treatment depends mainly on the underlying autoimmune disease. SLE myocarditis/AIC, although uncommon, is a serious complication, with a clinical prevalence of 9%. However, it was present in 57% of postmortem analyses, suggesting a high prevalence of subclinical disease [70]. Myocarditis is a rare but fatal complication of SLE. High-dose corticosteroid treatment is the mainstream medication [71,72] and is usually effective as initial treatment, leading to amelioration of left ventricular function. However, there are few cases presenting as cardiogenic shock necessitating mechanical support. Other drugs used in the treatment of SLE myocarditis/AIC are azathioprine, cyclophosphamide, rituximab, and intravenous immunoglobulins [73]. The interleukin-1 receptor antagonist anakinra has been successfully used in myocarditis/AIC during Still’s disease [74].

Vasculitis-induced AIC, apart from cardiac supportive medication, should be also treated with novel biologics, such as abatacept, tocilizumab, and ustekinumab, that offer more precise treatment in large-vessel vasculitis and promise to minimize the glucocorticoid dose. Novel therapies for B-cell in antineutrophil cytoplasmic antibody (ANCA)–associated vasculitis are based on rituximab. Finally, mepolizumab demonstrated the key role of interleukin-5 in eosinophilic granulomatosis with polyangiitis [75].

In SSc patients who develop myocarditis/AIC, timely initiation of immunosuppressive treatment, such as cyclophosphamide and corticosteroids [76], in parallel with adequate cardiac medication, can delay the VT development and the progression of cardiac dysfunction. It is possible that a subclinical but impaired myocardial performance can be improved by moderate corticosteroid doses in the short-term [77], but this should be further confirmed by double-blind controlled studies.

**Figure 3:**
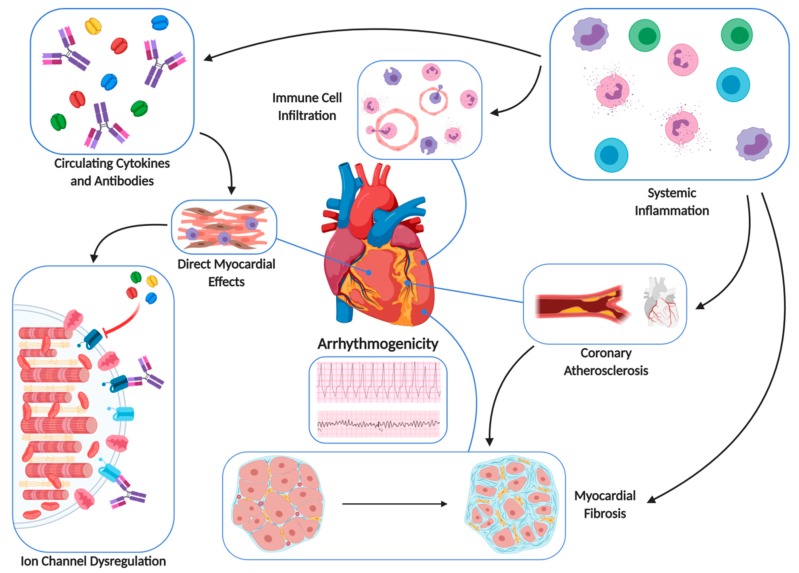
A schematic presentation of the interaction between cardiology and rheumatology in the development of AIC.

A schematic presentation of the interaction between cardiology and rheumatology in the development of AIC is presented in Figure 3.

## 8. Conclusions

AIC in ARDs is a recently recognized entity that can explain the presence of VA/SCD in ARDs without coronary artery disease. Clinical assessment and evaluation with ECHO-GLS, PET, and CMR can facilitate both early diagnosis, effective treatment, and follow up. AIC treatment should include a combination of cardiac and antirheumatic medication. However, in life-threatening cases, circulatory support with external devices, and/or implantable pacemakers/defibrillators can be life-saving.

## Figures and Tables

**Table 1:** Comparison between Echo, PET, and CMR in the evaluation of AIC.

	Spatial Resolution	RV Assessment	Radiation	Availability	Cost
Echo	+++	+	_	+++	+
PET	+	+	+++	+	+++
CMR	+++	+++	_	++	++
